# Prevalence of Intestinal Parasites and Associated Risk Factors among Diarrheal Patients Attending Negelle Borena General Hospital: A Case-Control Study

**DOI:** 10.1155/2023/1990468

**Published:** 2023-02-03

**Authors:** Zewdineh Firdu, Kucho Mulatu

**Affiliations:** ^1^Madda Walabu University (MWU), College of Natural and Computational Sciences, Department of Biology, Bale-Robe, Ethiopia; ^2^Negelle Secondary School (NSc) No. 2, Oromia Region, Negelle-Borena, Ethiopia

## Abstract

**Background:**

In tropical countries, intestinal protozoal parasitic infections are among the common infections causing significant morbidity and mortality. Thus, the present study was conducted to assess the status of intestinal protozoal parasitic infections among diarrheal patients attending Negelle Borena General Hospital and investigate the possible risk factors for the infection.

**Methods:**

A case-control study design was employed in the study. The intestinal protozoal parasites were detected using a wet-mount, stool concentration, and modified Ziehl–Neelsen methods. The crude and adjusted odd ratios were considered to identify the associated risk factors for intestinal protozoal parasitic infections among the study participants.

**Results:**

The overall status of parasitic infection was 46.88% in cases and 27.08% in the control groups. The most prevalent protozoal infection was *Giardia lamblia* (9.38%) and followed by *Entamoeba histolytica/dispar* (6.25%) in the cases. The highest prevalence of the infection was detected in the age groups that varied from 6 to 10 years in cases (71%). The enteric *G. lamblia* and *E. histolytica/dispar* were found to be 33.3% and 9.1%, respectively, in the age groups of 6–10 years in cases. Based on sex, 56.50% and 27.30% of protozoal (parasitic) infections were revealed by females in the cases and control groups, respectively, indicating a significant variation (*P* < 0.05). The education level (illiteracy), absence of toilet usage, no hand washing after toilet and before eating as significant risk factors for patient's infection with *G. lamblia*, *E. histolytica*, and *Cryptosporidium* spp. (*P* < 0.05, AOR = 1–14). However, eating raw fruit and vegetables was also found as a major risk factor for *E. histolytica* (*P* < 0.05, AOR = 6.2) 40. Moreover, the residence of the patients was also indicated as a plus risk factors for the infection to be occurred by *Cryptosporidium* spp. in the study participants (*P* > 0.95, AOR = 0.2).

**Conclusion:**

The prevalence of intestinal protozoal parasitic infection showed variation based on age, sex, and educational background of the study participants. Therefore, awareness creating training should be provided to the Negelle area communities so as to minimize the parasitic infection.

## 1. Background

In tropical countries, intestinal protozoan infections are among the common infections causing significant morbidity and mortality [1]. The etiological agent of Giardiasis, *Giardia duodenalis* (*Giardia intestinalis*, *Giardia lamblia*), is one of the most prevalent intestinal protozoan flagellate of the human. The life cycle of *Giardia* species includes active trophozoite and cyst that transmit via fecal-oral route. *Giardia intestinalis* is a frequent cause of diarrhea, affecting approximately 200 million people worldwide [2]. Worldwide, approximately 40 million people suffer annually with infection of *Entamoeba histolytica* and 40,000 die due to the resulting dysentery and liver abscesses [3]. It is also estimated that *E. histolytica* kills between 40,000 and 100,000 people per year and considered as one of the deadliest parasitic infections worldwide [4], whereas *Cryptosporidium* spp. is primarily affecting immunocompromised patients like HIV/AIDS patients [5].

The distribution and prevalence of intestinal parasites differ from country to country and even regionally within countries because of several environmental, social, and geographical factors, which are the major risk factors in the transmission of parasitic infections, especially protozoan infections. In this aspect, age, sex, poor sanitation, source of drinking water, personal hygiene, location, contact with animals, and seasonal variations are the most widely reported risk factors for protozoal parasitic infection in human beings [6].

Intestinal parasites are widely distributed in Ethiopia due to lack of environmental and personal sanitation, contamination of food and drinking water [7, 8]. According to the Ethiopian Ministry of Health, more than 250,000, annual visit of outpatient services of the health institutions is due to intestinal parasitic infections (IPIs) [1]. In addition, different reports have been reported from several parts of Ethiopia such as 72.9% in Gondar (Azezo), 83% in Jimma, and 83.8% in South East of Lake Langaano [9–11]. There is a scanty of study conducted among individuals affected by diarrheal diseases attending Negelle Borena General Hospital. Thus, the main objective of this study was to assess the prevalence of intestinal protozoan parasitic infection in the hospital and determine some associated risk factors.

## 2. Materials and Methods

### 2.1. Description of the Study Area

This study was conducted in Negelle town at Negelle Borena General Hospital in Guji zone Oromia region, southern Ethiopia. The town is located at a distance of 595 km far from Finfine (Addis Ababa), the capital city of Ethiopia. Geographically, the town is located between 4°30′58″–5°42′8″ latitudes and 41°34′57″–39°9′34″ altitude.

### 2.2. Description of Study Population and Exclusion Criteria

In the present study, individuals with diarrheal and abdominal discomfort were considered as a case group, and those without diarrheal and abdominal discomfort were taken as a control group. As exclusion criteria, patients who received anti-protozoal and parasitic medication for the last 3 months were excluded from the study. A case-control study design was also employed during the study time. A case-control study is defined as a study that compares two existing groups as, for example, disease vs. no disease based on the observed characteristics of the groups.

### 2.3. Sample Size Determination

The sample size was calculated by using single proportion formula by considering the number of patients who visited Negelle General Hospital during the study period. The sample size of the study participant was calculated according to Jamal [12].

### 2.4. Stool Sample Collection and Processing

A single stool specimen was collected from each participant. Freshly voided stool specimen (Appx. 0.5 mg) was directly examined microscopically and the remaining was preserved with 10% formalin for further parasitic analysis using the stool concentration and modified Ziehl–Neelsen methods as described below.

### 2.5. Methods of Parasitological Study

#### 2.5.1. Wet-Mount

A direct wet-mount of stool specimen was conducted according to Cheesbrough [13]. The stool specimens were obtained from all study participants. Then a direct saline wet-mount microscopy of each sample was used to detect intestinal protozoal parasites microscopically. In this method, one drop of physiological saline was added on a clean slide and then a stool equivalent to a match stick head (2 mg) was mixed with it, and was examined under low power (10×) and high power (40×) objectives, respectively, to examine at the structures (the trophozoites stages) and determine the type of protozoa (parasites) found in the patient's stool samples.

#### 2.5.2. Stool Concentration

A portion of each preserved stool specimen was taken using an applicator stick and processed following standard procedures. In this concentration procedure, one gram (1 g) of stool was placed in a clean conical centrifuge tube containing 7 mL 10% formol-water by using applicator stick. The resulting suspension was then filtered through a sieve into another conical tube. After adding 3–4 mL of diethyl-ether to the suspension, the content was centrifuged at 3200 rpm for 1 minute. The supernatant was discarded, the smear was prepared from the sediment and observed under light microscope with a magnification of 10× and 40× once air dried as described by Cheesbrough [13].

#### 2.5.3. Modified Ziehl–Neelsen Method

The stool specimens were analyzed using modified Ziehl–Neelsen method as described by Tahvildar-Biderouni and Salehi [14]. Stool specimens were prepared to smear from the concentrated stool samples, air dried, fixed by ethanol, later alkaline fusion was poured on the slides and heated until it brought up to steam, but not boiled, after 5 minutes, slides were washed and decolorized by 2.5% sulfuric acid for 1 minute, depending on the film thickness then counterstained with 1% methylene blue for 1 min then washed and air dried and examined with 100× objective.

### 2.6. Identification of Protozoan Parasites

The identification of protozoan pathogens was based upon direct detection of cysts, oocysts, eggs, and larval stages of the parasites in the stool concentration and modified Ziehl–Neelsen methods using a compound microscope. The genera and/or species determination of the protozoan was done by the experienced laboratory technicians working in the health centers.

### 2.7. Quality Control

In order to be certain with the quality of our data, 5% of the total positive specimens in our current study were randomly selected and re-examined by experienced laboratory technicians who did not have any information about the previous results. Moreover, for the quality checkup purpose, the control slides were also used during each run of the stain examination.

### 2.8. Methods of Data Collection, Processing, and Analysis

The questionnaires were prepared in English language and then translated to Amharic and Afan Oromo version during the interview as appropriate. The questionnaires were filled by the study participants with the help of the investigator and nurses working in the hospital. Having completed the data gathering from the interviewee, the questionnaires were checked for their proper filling. The univariate and multivariate analysis was performed using SPSS version 21 statistical software. The crude and adjusted odd ratios were also considered to identify the associated risk factors for intestinal protozoal parasitic infection of the study participants. All the significant values were considered at *P* < 0.05 throughout the analysis.

## 3. Results

### 3.1. Socio-Demographic Characteristics of the Cases and Control Groups

In the present study, among the cases,100 (52.08%) and 92 (48.00%) were males and females, respectively. In the same manner, 82 (43.00%) and 110 (57.30%) were males and females in the control groups, respectively. The age distribution indicated that majority of the study participants were in the age groups of 6–10 years, which was 40.63% and 29.69% in cases and the control groups, respectively. On the other side, 62.50% of the patients who were found to have diarrheal diseases and abdominal discomforts (case groups) are confirmed to live in the rural area. The educational status (Grade 1–6) of about 40.63% and 32.29% was also notified in the cases and control groups, respectively. Occupationally, most of the cases were private workers (40.63%), whereas 31.25% of the control groups were non-governmental employees (Table 1).

### 3.2. Intestinal Protozoal Parasitic Infection in Cases and Control Groups

Microscopic examination of stool for the presence of any protozoal parasite was done using wet-mount, stool concentration, and modified Ziehl–Neelsen methods in both groups (i.e., the cases and controls). The parasitological methods indicated *Ascaris lumbricoides*, *E. histolytica/dispar*, *G. lamblia*, *Schistosoma mansoni*, *Hook worm*, *Taenia* spp., *Cryptosporidium* spp., *Strongyloid stercolaris*, *Trichuris trichiura*, and *Hymenolepis nana* as the major parasites found to infect individuals with abdominal discomfort and diarrheal disease (cases) and none diarrheal disease and abdominal problematic individuals (the controls). The overall status of parasitic infection was 46.88% in cases and 27.08% in the control group. Of the IPI, *E. histolytica/dispar* is found to co-infect individuals being with *G. lamblia* and *A. lumbricoides* as double infection. *Giardia lamblia* is also found as double infection together with *Cryptosporidium* spp. In this regard, about 8.33% and 4.17% of the study, participants were positive for the double infection in the cases and control groups, respectively (Table 2).

The most prevalent protozoal (parasitic) infection was *G. lamblia* (9.38%), which was followed by *E. histolytica/dispar* (6.25%) in the cases. *Schistosoma mansoni* was relatively the highest infectious parasite (4.69%) in the control groups being followed by *G. lamblia* (3.65%) and *E. histolytica/dispar* (2.08%). *Cryptosporidium* spp. and *S. stercolaris* were also found dominant in the case groups with relatively highest prevalence as compared with control groups.

### 3.3. Prevalence of Intestinal Protozoal (Parasites) across Age Groups (Years) of the Study Participants

In the present study, the total variation of parasitic infection was assessed based on age wise of different category (Table 3). As indicated in this result section, the highest prevalence of the infection was displayed by the age groups that varied from 6 to 10 years in cases (71%). However, in the control groups, the highest prevalence was noted in the groups of 11–20 years (63%). The enteric *G. lamblia* and *E. histolytica/dispar* were found to be 33.3% and 9.1%, respectively, in the age groups of 6–10 years in cases, which might be associated with abdominal discomfort in the studied populations. In addition, the lower status of parasitic infection was exhibited by the age groups >41 years in cases (37.00%), <5 years (3%), and 14% in >41 years in the control groups.

### 3.4. Prevalence of Intestinal Protozoal (Parasites) of the Study Participants Based on Sex

The prevalence of intestinal parasitic infection indicated variation among the study population based on the sex of the participants. As shown in Table 4, the highest infection was observed in females rather than in male in both the study groups. In this regard, 56.50% and 27.30% of protozoal (parasitic) infections were revealed by females in the cases and control groups, respectively. On the other hand, the statistical analysis indicated a significant variation (*P* < 0.05) of infection based on sex in both groups (cases and control groups). In cases, the co-infection of *E. histolytica/dispar* and *G. lamblia* was shown in females (8.7%) than in the males.

### 3.5. Univariate and Multivariate Logistic Regression Analysis of Associated Risk Factors for Protozoal (Parasitic) Infections of the Study Participants

#### 3.5.1. Univariate Logistic Regression Analysis

The univariate logistic regression was conducted to evaluate the possible risk factors that are responsible for the intestinal protozoal parasites among and/or between the study participants as shown in Table 5. The analysis indicated that having contact with pet animals, residency, associated health problems, and source of drinking water with P values of greater than 0.25, which is the cut point of the univariate logistic regression analysis for *G. lamblia*. For *E. histolytica*, level of education, source of drinking water, washing hand with soap before eating, associated health problems, and having contact with pet animals were not found significant (i.e., with a value of greater than 0.25). However, it was eating raw fruit and vegetables and the permanent residence that showed a P value of greater than 0.25 for the *Cryptosporidium* spp.

#### 3.5.2. Multivariate Logistic Regression Analysis

The multivariate analysis indicated the illiteracy of participants and mothers, the absence of toilet utilization, no hand washing after toilet and before eating as significant risk factors (*P* < 0.05) for patient's infection with *G. lamblia*, *E. histolytica*, and *Cryptosporidium* spp. However, eating raw fruit and vegetables was also found as a major risk factor for *E. histolytica* (*P* < 0.05, AOR = 6.2) 40. Moreover, the residence of the patients was also indicated as a plus risk factor for the infection to be occurred by *Cryptosporidium* spp. in the study participants (*P* > 0.05, AOR = 0.2) (Table 6).

## 4. Discussion

The current study assessed the prevalence of intestinal parasites among patients visiting Negelle Borena General Hospital. The results from stool sample analysis using microscope examinations indicated that the overall prevalence of IPIs was 46.88% in cases and 27.08% in the control groups. In the case groups, the finding of this study was higher than prevalence of parasites at Workmeda Health Center, Ethiopia, 27.7%, Uganda, 32.8%, Nigeria, 41.2% [15, 16]. On the other hand, this study had showed a lower prevalence of parasitic infection than the reports in Southwest Ethiopia (83%), Tseda Health Center, Northwest Ethiopia, 62.2% [17]. This indicates that there is variation of parasitic infection from place to place and country to country. The variations might be due to the difference in the characteristics of the study population, geographical distribution, and diagnostic techniques used in this and other studies.

The comparison made through the statistical analysis between the cases (abdominal discomfort and diarrheal disease) and the control group (none diarrheal disease and abdominal problematic individuals) showed a significant variation (*P* < 0.05), whereby the highest stool positives were found in the cases group. This may indicate as the enteric protozoan and parasites are responsible merely for the occurrence of diarrhea and abdominal discomfort in human being (study participants of this study too). It has been reported that intestinal protozoan and parasites showed the highest prevalence in patients with gastrointestinal discomforts and are related to diseases such as diarrhea [18]. On the other side, more than 50% of double infection was observed in the cases than in the control groups. In the case group, it was the *E*. *histolytica/dispar* and *G. lamblia* that mainly involved in the co-infections observed in the present study. Thus, this might be associated with the diversity and ability of these protozoan pathogens to initiate gastrointestinal disorder in the group. The most important protozoan etiological agents of IPIs are *E. histolytica/dispar* (affecting 50 million people) and *G. lamblia* (affecting 200 million people) [19]. *Cryptosporidium* spp. and *S. stercolaris* were also found dominant in the case groups with relatively showing the highest prevalence as compared with control groups.

The age-wise prevalence of intestinal protozoan (parasites) indicated that the highest results were found in the age group of 6–10 years (71%). This finding is in line with the finding reported by Al-Jawabreh et al. [20], whereby in children less than 14 years old, the infection rate was significantly higher (67%) than in adults and children (OR = 2.6, *P* = 0.038). This could be due to the independency of this age group from their parents and spend most of the time outside, they are highly interactive outdoor, thus this can enhance the probability of being infected by parasites. The same result was observed in the control groups with lowest infection in the age group of greater than 41 years (14%). However, the highest infection was noted in the age group of 11–20 years (63%) in this group. The age-wise prevalence of IPI revealed reduction in the infection level as the age lies above 10 years old. This is in agreement with the study conducted by Al-Jawabreh et al. [20]. The second higher infection of parasitic infection was also noted in the age groups of less than 5 years old, which was 61% and 3% in cases and control groups, respectively. However, the minimum prevalence in the age groups greater than 41 years was 37% in the case groups and 14% in the controls. This finding is supported by Mohammed [21] who reported less prevalence intestinal parasitic infection in age group of 41–50 years old (47.6%). This might be associated with age of the individuals in the group since majority of them could be the guardian of children and family owner, they are responsible for their child sanitation and feeding, which can contribute for their safety indirectly. Besides, lower rate in this age group can be explained by assuming perhaps their good mastery and awareness of hygiene and sanitation practices, combined with sedentary state of life style.

In the present study, the prevalence of intestinal protozoan and parasites was assessed based on the gender of the study participants. The result pointed out a significant variation (*P* < 0.05) of parasitic infection in the cases between male and female of study participants. In this aspect, the prevalence was 56.5% and 37% in females and males, respectively. This may be an indication that the two genders are not equally exposed to infection. The highest infection of females by intestinal parasites found in the present study is not in agreement with the finding reported by Mohammed [21], in which intestinal parasites were more prevalent between the male patients (65.7%) than the females (57.4%). Nevertheless, this finding is in line with Acharya et al. [22] whose study indicated the highest prevalence of parasitic infection in females (32.11%) as compared with the males (21.99%). Moreover, Patel et al. [23] and Zemene and Shiferaw [24] reported similar results in their study with predominance of parasitic infection in females. In Ethiopia, females are mostly involved with child care in home, house cleaning, processing of raw fruits and vegetables, cleaning of home compound and clothes more than males, thus they are comparatively more exposed to contaminated soil and water, a major predisposing factor for the infection in the females.

The suspected (associated) risk factors for intestinal protozoal parasitic infection in the case groups were analyzed using univariate and multivariate logistic regression analysis. As presented in the result section, the multivariate analysis indicated the illiteracy of participants and mothers, the absence of toilet utilization, no hand washing after toilet and before eating as significant risk factors for patient's infection with *G. lamblia*, *E. histolytica*, and *Cryptosporidium* spp. (*P* < 0.05, AOR = 1–14). However, eating raw fruit and vegetables was also found as a major risk factor for *E. histolytica* (*P* < 0.05, AOR = 6.2) 40. Moreover, the residence of the patients was also indicated as a plus risk factors for the infection to be occurred by *Cryptosporidium* spp. in the study participants (*P* > 0.95, AOR = 0.2). This may indicate as the educational status of any person can determine the health of the individuals. Because as people are more educated, the more they take care of their own safety in terms of hygiene and sanitation, conditions that are basic for the prevention of parasitic infection. Similarly, the mother's education is a basis for the prevention of parasitic infection. Because the role of mothers is immense in the family leading, managing children, cooking, and other several activities such as following children's activity most of the time in the Ethiopian context.

The permanent residence can also have an effect for the individuals to be infected by parasitic infections. Mostly, individuals living in rural area are involved in the agricultural activity (crop cultivation and animal rearing), a situation which exposes them to have contact with soils, animals, and animal wastes such as dungs. This is an indication for the association of *Cryptosporidium* spp. with pet animals as a significant risk factor for the study participants to be infected with it. Cryptosporidiosis is a highly prevalent gastrointestinal parasitic disease caused by protozoan species of the genus *Cryptosporidium* that infect a wide range of animals, including people, throughout the world. *Cryptosporidium parvum* is also a common enteric infection in young lambs and goats. Diarrhea can result from a monoinfection but more commonly is associated with mixed infections. Infection can be associated with severe outbreaks of diarrhea, with high case fatality rates in lambs 4–10 days old and in goat kids 5–21 days old [25].

## 5. Limitations of This Study

There were some limitations in this study. Firstly, stool samples were collected only once, so that it is likely that the analysis of three consecutive stool samples could have increased the number of protozoal parasites to be identified in the study. Second, there is a possibility that some of the participants have decided to take anti-parasitic drugs prior to the stool sampling, considering that metronidazole, mebendazole, and albendazole can be purchased without prescription in Ethiopia, which can even reduce the number of positive stool specimen. Thirdly, it is likely that the prevalence of some protozoa such as *E. histolytica/dispar* could be even higher if culture techniques had been used.

## 6. Conclusion

The prevalence of intestinal protozoal parasitic infection showed variation based on age, sex, and educational background of the study participants. Therefore, awareness creating training should be provided to the Negelle area communities so as to minimize the parasitic infection.

## Figures and Tables

**Table 1:** Socio-demographic distribution of cases (*N* = 192) and controls (*N* = 192) in a study of intestinal protozoal parasitic infections among patients visiting Negelle Borena General Hospital.

Variables	Options	Cases *N* (%)	Control *N* (%)	Total *N* (%)
Sex	Male	100 (52.08)	82 (43.00)	182 (47.39)
	Female	92 (48.00)	110 (57.30)	202 (52.60)
Age (year)	<5	54 (28.13)	30 (15.63)	84 (21.88)
	6–10	78 (40.63)	57 (29.69)	135 (35.16)
	11–20	30 (15.63)	20 (10.42)	50 (13.02)
	21–30	20 (10.42)	25 (13.02)	45 (11.72)
	31–40	6 (3.13)	29 (15.10)	35 (9.11)
	Above 40	4 (2.08)	31 (16.15)	35 (9.11)
Residence	Urban	72 (37.50)	85 (44.27)	157 (40.89)
	Rural	120 (62.50)	107 (55.73)	227 (59.11)
Educational level	Illiterate	8 (4.17)	14 (7.29)	22 (5.73)
	Kindergarten	58 (30.21)	47 (24.48)	105 (27.34)
	Grade 1–6	78 (40.63)	62 (32.29)	140 (36.46)
	Grade 7–12	30 (15.63)	48 (25)	78 (20.31)
	Above grade 12	18 (9.38)	21 (10.94)	39 (10.16)
Occupation	Government employee	42 (21.88)	43 (22.40)	85 (22.14)
	Private	78 (40.63)	32 (16.67)	110 (28.65)
	NGO employee	28 (14.58)	60 (31.25)	88 (22.92)
	Trade	44 (22.92)	57 (29.69)	101 (26.30)

*N* = Total number of the study participant in cases and control groups.

**Table 2:** Prevalence of intestinal protozoan and parasites in cases (*N* = 192) and control (*N* = 192) among individuals visiting Negelle Borena General Hospital.

Type of protozoa (parasites)	Cases (*N* (%))	Control (*N* (%))	Difference	*P*-value
*Ascaris lumbricoides*	7 (3.65)	2 (1.04)	2.65	0.091
*Entamoeba histolytica/dispar*	12 (6.25)	4 (2.08)	4.17	0.028∗
*Giardia lamblia*	18 (9.38)	7 (3.65)	5.73	0.022∗
*Schistosoma mansoni*	6 (3.13)	9 (4.69)	1.56	0.430
*Hook worm*	9 (4.69)	6 (3.13)	1.56	0.430
*Taenia* spp.	3 (1.56)	4 (2.08)	0.52	0.703
*Cryptosporidium* spp.	4 (2.08)	0 (0.00)	2.08	0.031∗
*Strongyloid stercolaris*	4 (2.08)	1 (0.52)	1.56	0.177
*Trichuris trichiura*	3 (1.56)	5 (2.60)	1.04	0.475
*Hymenolepis nana*	8 (4.17)	6 (3.13)	1.04	0.586
*E. hist./dispar* + *G. lamblia*	9 (4.69)	4 (2.08)	2.61	0.157
*Cryptosporidium* spp. + *G. lamblia*	3 (1.56)	1 (0.52)	1.04	0.315
*E. hist./dispar* + *A. lumbricoides*	4 (2.08)	3 (1.56)	0.52	0.703
**Total**	**90 (46.88)**	**52 (27.08)**	**19.80**	**≤0.001∗**

*N* = Total number of the study participant in both cases and the control groups, ∗statistically significant (*P* < 0.05).

**Table 3:** Prevalence of intestinal protozoal parasites based on age (years) among diarrheal patients (cases) attending Negelle Borena General Hospital.

Type of protozoa (parasites)	Cases age (years)						Control age (years)					
	<5 (*N* = 18)	6–10 (*N* = 14)	11–20 (N =20)	21–30 (*N* = 55)	31–40 (*N* = 25)	>41 (*N* = 60)	<5 (*N* = 32)	6–10 (*N* = 45)	11–20 (*N* = 30)	21–30 (*N* = 48)	31–40 (*N* = 30)	>41 (*N* = 27)
*A. lumbricoides*	—	—	—	—	5 (20)	2 (3.3)	1 (3.12)	1 (2.22)	—	—	—	—
*E. histolytica/dispar*	—	1 (7.1)	1 (5)	5 (9.1)	—	3 (5)	—	2 (4.4)	1 (6.7)	1 (10)	—	—
*G. lamblia*	—	—	2 (10)	8 (33.3)	2 (8)	6 (10)	—	—	2 (13.3)	4 (40)	1 (1.3)	—
*S. mansoni*	—	—	1 (5)	3 (5.5)	—	2 (3.3)	—	3 (6.7)	3 (2.2)	3 (30)	—	—
*H. worm*	1 (5.56)	2 (14.3)	1 (5)	2 (3.6)	3 (12)	—	—	3 (6.7)	2 (3.13)	1 (10)	—	—
*Tenia* spp.	2 (11.11)	1 (7.1)	—	—	—	—	—	—	3 (2.2)	—	1 (1.3)	—
*Cryptosporidium* spp.	2 (11.11)	2 (14.3)	—	—	—	—	—	—	—	—	1 (1.3)	—
*S. stercolaris*	4 (22.22)	—	—	—	—	—	—	—	—	—	—	—
*T. trichiura*	2 (11.11)	1 (7.1)	—	—	—	—	—	—	1 (6.7)	1 (10)	3 (3.8)	—
*H. nana*	—	3 (21.4)	1 (5)	2 (3.6)	—	3 (5)	—	2 (4.4)	4 (26.7)	1 (10)	—	—
*E. hist./dispar* and *G. lamblia*	—	—	3 (15)	4 (7.3)	—	2 (3.3)	—	—	—	2 (20)	—	2 (16.7)
*Cryptosporidium* spp*. + G. lamblia*	—	—	3 (15)	—	—	—	—	—	—	—	—	1 (8.3)
*E. hist./dispar* + *A. lumbricoides*	—	—	—	—	—	4 (6.7)	—	—	—	—	2 (2.6)	1 (8.3)
**Total**	**11 (61)**	**10 (71)**	**12 (60)**	**24 (44)**	**10 (40)**	**22 (37)**	**1** (3)	**11** (24)	**19** (63)	**13** (46)	**8** (27)	**4** (14)

**Table 4:** Prevalence of intestinal protozoal parasites based on sex among diarrheal patients (cases and controls) visiting Negelle Borena General Hospital.

S. N	Parasitic infection	Cases (*N* = 192)		*p-value*	Controls (*N* = 192)		*p*-value
		Female (*N* = 92) *n* (%)	Males (*N* = 100) *n* (%)		Female (*N* = 110) *n* (%)	Male (*N* = 82) n (%)	
1	*A. lumbricoides*	4 (4.3)	3 (3.0)	0.630	2 (1.8)	0 (0.00)	0.258
2	*E. histolytica/dispar*	9 (9.9)	3 (3.0)	0.049^a^	3 (2.7)	1 (1.2)	0.469
3	*G. lamblia*	14 (15.2)	4 (4.0)	0.007^a^	5 (4.5)	2 (2.4)	0.439
4	*S. mansoni*	2 (2.2)	4 (4.0)	0.474	4 (3.6)	5 (6.1)	0.417
5	*H. worm*	4 (4.3)	5 (5.0)	0.818	3 (2.7)	3 (3.7)	0.693
6	*Tenia* spp.	1 (1.1)	2 (2.0)	0.616	1 (0.9)	3 (3.7)	0.180
7	*Cryptosporidium* spp.	2 (2.2)	2 (2.0)	0.990	1 (0.9)	1 (1.2)	8.837
8	*S. stercolaris*	1 (1.1)	3 (3.0)	0.357	0 (0.0)	0 (0.0)	0.990
9	*T. trichiura*	1 (1.1)	2 (2.0)	0.616	2 (1.8)	3 (3.7)	0.414
10	*H. nana*	3 (3.3)	5 (5.0)	0.556	4 (3.6)	2 (2.4)	0.634
11	*E. hist/dispar*. and *G. lamblia*	8 (8.7)	1 (1.0)	0.011^a^	3 (2.7)	1 (1.2)	0.645
12	*Cryptosporidium* spp. and *G. lamblia*	1 (1.1)	2 (2.0)	0.616	1 (0.9)	0 (0.0)	0.252
13	*E. hist./dispar* and *A. lumbricoides*	2 (2.2)	1 (1.0)	0.504	2 (1.8)	1 (1.2)	0.738
**Total**		**52 (56.5)**	**37 (37.0)**	**0.006**	**31 (28.2)**	**22 (26.8)**	**0.938**

^a^Statistically significant (*P* < 0.05).

**Table 5:** Univariate logistic regression analysis for the associated risk factors of intestinal protozoal parasitic infection among positive diarrheal patients (cases) attending Negelle General Hospital.

Risk factors	Options	*G. lamblia n* (%)	cOR	*p-*value	*E. Hist./dispar n* (%)	cOR	*p*-value	*Cryptosp.* Spp. *n* (%)	cOR	*p-*value
Level of education	Illiterate	4 (28.6)	4.5	0.03^a^	1 (11.1)	0.25	0.42	0 (0.0)	—	—
	Kindergarten	1 (7.1)	1.9	0.16	0 (0.0)	0.25	0.42	0 (0.0)	—	—
	Grade 1–6	3 (21.4)	1.5	0.20	5 (55.5)	0.99	≤0.001^a^	1 (100)	0.8	0.13
	Grade 7–12	2 (14.3)	1.2	0.25	3 (33.3)	0.99	≤0.001^a^	0 (0.0)	—	—
	> Grade 12 (ref.)	4 (28.6)	—	—	0 (0.0)	—	—	0 (0.0)	—	—
Mothers educational level	Illiterate	6 (42.9)	3.7	0.01^a^	6 (66.7)	0.11	0.19	1 (100)	1.5	0.22
	Grade 1–12	1 (7.1)	2.8	0.09	3 (33.3)	0.17	0.29	0 (0.0)	—	—
	Above 12 (ref.)	7 (50.0)	—	—	0 (0.0)	—	—	0 (0.0)	—	—
Source of drinking water	Pipe	3 (21.4)	1.7	0.41	0 (0.00)	0.75	0.85	0 (0.0)	—	—
	Well	9 (64.3)	10	≤0.001^a^	8 (88.9)	3.0	0.47	1 (100)	0.5	0.06
	Packed (ref.)	2 (14.3)	—	—	1 (11.1)	—	—	0 (0.0)	—	—
Presence and use of toilet	Yes	4 (28.6)	0.2	0.16	4 (44.4)	0.13	0.14	0 (0.0)	—	—
	No (ref.)	10 (71.4)	—	—	5 (55.5)	—	—	1 (100)	7.2	≤0.001^a^
Washing hand with soap after toilet	Yes	7 (50.0)	0.80	0.09	5 (55.5)	0.13	0.14	0 (0.0)	—	—
	No (ref.)	7 (50.0)	—	—	4 (44.4)	—	—	1 (100)	1.8	0.05
Washing hand with soap before eating	Yes	5 (35.7)	1.0	0.03^a^	3 (33.3)	0.25	0.31	0 (0.0)	—	—
	No (ref.)	9 (64.3)	—	—	6 (66.7)	—	—	1 (100)	2.5	0.04^a^
Eating raw fruit and vegetables	Yes	12 (85.7)	0.17	0.13	7 (77.8)	0.13	0.16	0.00	—	—
	No (ref.)	2 (14.3)	—	—	2 (22.2)	—	—	1 (100)	1.8	0.88
Associated health problems	HIV (ref.)	0 (0.0)	—	—	0 (0.0)	—	—	0 (0)	—	—
	TB	14 (100)	0.33	0.40	9 (100)	0.83	0.76	1 (100)	7.2	≤0.001^a^
Contact with pet animals	Yes	6 (42.9)	1.7	0.64	6 (66.7)	0.33	0.38	1 (100)	7.2	≤0.001^a^
	No (ref.)	8 (57.1)	—	—	3 (33.3)	—	—	0 (0.0)	—	—
Residence	Rural	10 (71.4)	3.0	0.40	7 (77.8)	0.13	0.15	0 (0.0)	—	—
	Urban (ref.)	4 (28.6)	—	—	2 (22.2)	—	—	1 (100)	0.9	0.78

*n* = Number of individuals found positive for the parasites found in the stool sample of the patients. cOR, crude odd ratios.

^a^Statistically significant factors (*P* < 0.05).

**Table 6:** Multivariate logistic regression analysis for the associated risk factors of intestinal protozoal parasitic infection among positive diarrheal patients (cases) attending Negelle General Hospital.

Risk factors	Options	*G. lamblia n* (%)	AOR	*p-*value	*E. Hist./dispar n* (%)	AOR	*p-*value	*Cryptosp.* Spp *n* (%)	AOR	*p-*value
Level of education	Illiterate	4 (28.6)	2.3	0.01^a^	1 (11.1)	5.2	0.01^a^	0 (0.0)	0.6	0.52
	Kindergarten	1 (7.1)	1.8	0.19	0 (0.0)	1.6	0.2	0 (0.0)	0.6	0.52
	Grade 1–6	3 (21.4)	0.8	0.36	5 (55.5)	0.5	0.1	1 (100)	3.2	≤0.001^a^
	Grade 7–12	2 (14.3)	2.9	0.09	3 (33.3)	0.5	0.4	0 (0.0)	0.6	0.52
	> Grade 12 (ref.)	4 (28.6)	—	—	0 (0.0)	—	—	0 (0.0)	—	—
Mothers educational level	Illiterate	6 (42.9)	1.0	0.03^a^	6 (66.7)	3.7	0.04^a^	1 (100)	3.2	≤0.001^a^
	Grade 1–12	1 (7.1)	2.9	.08	3 (33.3)	0.6	0.2	0 (0.0)	0.6	0.52
	Above 12 (ref.)	7 (50.0)	—	—	0 (0.0)	—	—	0 (0.0)	—	—
Presence and use of toilet	Yes	4 (28.6)	7.8	0.04^a^	4 (44.4)	3.7	0.04^a^	0 (0.0)	—	—
	No (ref.)	10 (71.4)	—	—	5 (55.5)	—	—	1 (100)	3.2	≤0.001^a^
Washing hand with soap after toilet	Yes	7 (50.0)	14	0.00^a^	5 (55.5)	3.9	0.05	0 (0.0)	—	—
	No (ref.)	7 (50.0)	—	—	4 (44.4)			1 (100)	3.2	≤0.001^a^
Washing hand with soap before eating	Yes	5 (35.7)	14	≤0.001^a^	3 (33.3)	5.2	0.01^a^	0 (0.0)	—	—
	No (ref.)	9 (64.3)	—	—	6 (66.7)			1 (100)	3.2	≤0.001^a^
Eating raw fruit and vegetables	Yes	12 (85.7)	2.3	0.06	7 (77.8)	6.2	≤0.001^a^	0.00	—	—
	No (ref.)	2 (14.3)	—	—	2 (22.2)	—	—	1 (100)	3.2	≤0.001^a^
Associated health problems	HIV (ref.)	0 (0.0)	—	—	0 (0.0)	—	—	0 (0)	—	—
	TB	14 (100)	0.33	0.40	9 (100)	0.83	0.76	1 (100)	3.2	≤0.001^a^
Contact with pet animals	Yes	6 (42.9)	1.7	0.64	6 (66.7)	0.33	0.38	1 (100)	3.2	≤0.001^a^
	No (ref.)	8 (57.1)	—	—	3 (33.3)	—	—	0 (0.0)	—	—
Residence	Rural	10 (71.4)	0.7	0.39	7 (77.8)	0.9	0.5	0 (0.0)	—	—
	Urban (ref.)	4 (28.6)	—	—	2 (22.2)	—	—	1 (100)	0.2	0.06

*n* **=** Number of individuals found positive for the parasites found in the stool sample of the patients. AOR, adjusted odd ratios.

^a^Statistically significant factors (*P* < 0.05).

## Data Availability

The data used for this study will be available upon request from the corresponding author.
